# A novel system-level approach using RNA-sequencing data identifies *miR-30-5p* and *miR-142a-5p* as key regulators of apoptosis in myocardial infarction

**DOI:** 10.1038/s41598-018-33020-x

**Published:** 2018-10-02

**Authors:** Jin Ock Kim, Jei Hyoung Park, Taeyong Kim, Seong Eui Hong, Ji Young Lee, Kyoung Jin Nho, Chunghee Cho, Yong Sook Kim, Wan Seok Kang, Youngkeun Ahn, Do Han Kim

**Affiliations:** 1 School of Life Sciences and Systems Biology Research Center, Gwangju Institute of Science and Technology (GIST), Gwangju, 61005 Korea; 2 Department of Cardiology, Chonnam National University Hospital, Gwangju, Korea

## Abstract

This study identified microRNAs involved in myocardial infarction (MI) through a novel system-level approach using RNA sequencing data in an MI mouse model. This approach involved the extraction of DEGs and DEmiRs from RNA-seq data in sham and MI samples and the subsequent selection of two miRNAs: *miR-30-5p* (family) and *miR-142a-5p*, which were downregulated and upregulated in MI, respectively. Gene Set Enrichment Analysis (GSEA) using the predicted targets of the two miRNAs suggested that apoptosis is an essential gene ontology (GO)-associated term. *In vitro* functional assays using neonatal rat ventricular myocytes (NRVMs) demonstrated that *miR-30-5p* is anti-apoptotic and *miR-142a-5p* is pro-apoptotic. Luciferase assays showed that the apoptotic genes, *Picalm* and *Skil*, and the anti-apoptotic genes, *Ghr* and *Kitl*, are direct targets of *miR-30-5p* and *miR-142a-5p*, respectively. siRNA studies verified the results of the luciferase assays for target validation. The results of the system-level high throughput approach identified a pair of functionally antagonistic miRNAs and their targets in MI. This study provides an in-depth analysis of the role of miRNAs in the pathogenesis of MI which could lead to the development of therapeutic tools. The system-level approach could be used to identify miRNAs involved in variety of other diseases.

## Introduction

Myocardial infarction (MI), a leading cause of death worldwide, is associated with a sudden loss of oxygen and nutrient supply to cardiac cells^1^. MI symptoms are usually severe and complicated by multiple cellular changes associated with inflammation, apoptosis, extracellular matrix remodelling, and impaired contractility. Numerous *in vitro* and *in vivo* studies have been conducted in an attempt to identify the underlying mechanisms for this deadly disease and to develop therapeutic tools to overcome the symptoms caused by MI.

MicroRNAs (miRNAs) are small non-coding RNAs, known to be negative regulators of gene expression by destabilizing target mRNAs or inhibiting their translation^2^. Because miRNAs bind their targets with imperfect sequence complementarity, a single miRNA is capable of orchestrating multiple target genes that often share the same biological pathways. Conversely, the expression of individual genes can be co-targeted by multiple miRNAs. Therefore, the identification of a group of miRNAs that exert synergistic or antagonistic effects on disease may have important therapeutic implications. However, systematic and system-level approaches to identify and evaluate multiple miRNAs and their targets working together in the particular biological pathways have not been fully explored.

Development of high-throughput omics technology has enabled us to generate multi-layer omics data such as differentially expressed genes (DEGs) and differentially expressed micro RNAs (DEmiRs) in various disease models. So far, most high-throughput studies on heart disease have been carried out using single layer-omics data which are limited in their ability to unravel the complexity of the molecular interactions between the different layers. To date, there are few examples of multi-layer integrative studies published on the topic of heart disease. Zhu X. *et al*. identified *miR-340* as a key miRNA contributing to the progression of heart failure due to dilated cardiomyopathy by combining the expression profiles of mRNA and miRNAs^3^. Through integrated analysis using miRNA and mRNA expression data, Wang *et al*. demonstrated that increased levels of *miR-146b-5p* mediates atrial fibrosis in patients with nonvalvular paroxysmal atrial fibrillation by targeting TIMP-4^4^. However, all of the aforementioned studies were based on microarray assays that may have resulted in non-specific hybridization, biases due to hybridization strength, low sensitivity, and the inability to identify novel genes or novel splicing events^5^.

In the present study, we performed RNA-sequencing for both mRNA and miRNA, a revolutionary alternative that overcomes the limitations of microarray methods^5^, to simultaneously profile the transcriptomes and miRomes at the early, middle, and end stages of MI. By using a two-layer omics data integration with a logical top-down approach, the miRNA-target networks implicated in the progression of MI were identified. Among them, *miR-30-5p* and *miR-142a-5p* were shown to be robust throughout the course of MI and were closely associated with apoptosis, suggesting their distinct role in the pathogenesis of MI. For the first time, we demonstrated that *miR-30-5p* is anti-apoptotic, while *miR-142a-5p* is pro-apoptotic, and the apoptotic genes, *Picalm* and *Skil*, are direct targets of *miR-30-5p*, and the anti-apoptotic genes, *Ghr* and *Kitl* are direct targets of *miR-142a-5p*.

The combinatorial treatment of *miR-30-5p* mimics and *miR-142a-5p* inhibitor was found to synergistically protect cardiomyocytes from apoptotic cell death during MI because the expression levels of the two miRNAs during MI were regulated in completely opposite ways with non-overlapping targets. Our novel system-level approach using HTP data may be used for the development of new tools in the treatment of MI’s fatal symptoms. In addition, RNA-Seq data could provide further insights into the pathogenesis involved in various other important diseases.

## Results

### Generation and assessment of MI mouse model

An MI mouse model was generated by ligature of LAD at 3 different stages (1D: early; 1 W: middle; 8 W: late). One day after induction of MI, the hearts appeared to be gradually hypertrophic, as evidenced by the increased left ventricular mass-to-body weight ratio (LVM/BW) (Supplementary Fig. 1A). LV fractional shortening (FS) and ejection fraction (EF) were significantly reduced in 1 W and 8 W after MI induction compared with sham group (*P* < 0.05) (Supplementary Fig. 1B,C). Molecular markers for MI gene expression such as *Nppa* and *Tlr2*^6^ were also significantly upregulated. (Supplementary Fig. 1D,E), suggesting severe cardiac remodelling post-MI.

### Deep sequencing reveals dynamic mRNA and miRNA signatures in mouse hearts post-MI

To generate differentially expressed mouse RNAs (DEGs) and miRNAs (DEmiRs) during the progression of MI, we performed deep RNA-seq and miRNA-seq for genome wide gene expression profiling. The mean numbers of the total mapped reads by RNA-seq and miRNA-seq were 33,951,175 and 21,532,321, and the overall read mapping ratios were 96.84% and 76.34%, respectively (Supplementary Tables 1 and 2). For the transcript expression quantification in RNA-seq, the number of reads for the genes were normalized to FPKM and for miRNA-seq, the normalized read counts for each annotated miRNA were presented as RPM (see “Materials and Methods” and Supplementary Fig. 2). Scatter plots of the normalized read counts of all mRNA (R^2^ > 0.99; Supplementary Fig. 3A) and miRNA (R^2^ > 0.99; Supplementary Fig. 3B) showed high degrees of correlation between biological replicates, indicating their high levels of reproducibility.

**Figure 1:**
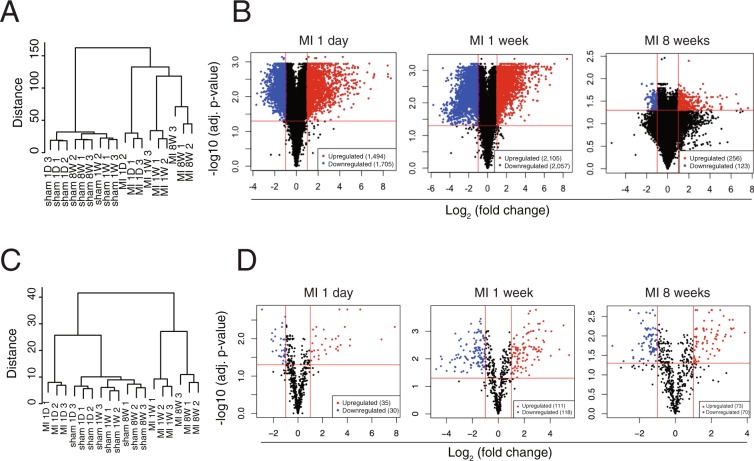
Differentially expressed genes in mouse model of MI derived by RNA-seq. (**A**) An unsupervised cluster analysis of the mRNA-seq data of MI and sham at 3 different stages. (**B**) Volcano plot showing DEGs in 1 day, 1 week, and 8 weeks post-MI. (**C**) An unsupervised cluster analysis of the miR-seq data of MI. (**D**) Volcano plot showing DEmiRs in MI. Red dots indicate significantly upregulated miRNAs and blue dots indicate downregulated miRNAs.

Unsupervised hierarchical clustering of the cardiac transcriptome and miRome revealed that both the mRNA and miRNA expression signature not only distinguished MI from the sham but also distinguished the 3 different stages of MI (Fig. 1A,C). Three-dimensional principal component analyses (PCA) also revealed that the mRNA expression profile clearly discriminates cardiac LV samples into 3 distinct stages post-MI, further indicating the existence of a distinct gene expression program during the course of MI (Supplementary Fig. 4).

After applying a stringent filtering approach (adjusted *p*-value < 0.05, |fold change| >2) that compared MI with sham, we identified 7,740 DEGs (early stage: 3,199, middle stage: 4,162, end stage 379) (Fig. 1B, red and blue coloured dots) and 437 DEmiRs (early stage: 65, middle stage: 229, end stage: 143) (Fig. 1D, red and blue coloured dots), respectively. Venn diagrams depict the overlap of DEGs at the 3 different stages of MI (Supplementary Fig. 5). Notably, the number of both DEGs and DEmiRs are dramatically reduced at the end stage of MI, suggesting severe myocardial cell death.

The top 50 most highly expressed RNAs and miRNAs at each stage of MI are presented in Supplementary Fig. 6. Strikingly, the percentage of the top 50 miRNA expression accounts for ~96% of the total miRNA expression (Supplementary Fig. 6B), demonstrating that only a subset of the miRNAs may play a central role in the pathogenesis of the heart. On the other hand, the percentage of the top 50 mRNA species is much lower compared with miRNA (less than 30%) (Supplementary Fig. 6A). Thus, a subset of miRNAs could regulate a number of mRNAs due to their multiple target binding sites as generally known, and only the 50 most highly expressed RNAs were focused in the present study.

### Gene Set Enrichment Analysis (GSEA) identifies critical pathways in the progression of MI

To identify the signalling pathways involved in the progression of MI (early, middle, and end stages) over the corresponding sham group, GSEA was performed on the whole set of expressed genes ranked by their differential expression in MI versus sham at 1 day, 1 week, and 8 weeks post-MI. Supplementary Fig. 7 shows the results of GSEA for each time point with hallmark gene sets from MSigDB using the GSEA tool. The results show that immune response-regulating signal transductions (e.g. TNFα signalling via NFKB, inflammatory response, IL6 JAK-STAT3 signalling, interferon alpha response, interferon gamma response, and IL2 STAT 5 signalling) were among the top over-represented pathways in the early stage of MI and were continuously activated until the middle stage of MI. Apoptosis signalling and fibrosis-related signalling such as TGF beta signalling and epithelial mesenchymal transition (EMT) were also significantly activated in the onset of MI and lasted until the middle stage of MI. Meanwhile, genetic programs such as fatty acid metabolism and oxidative phosphorylation were significantly downregulated in the early and middle stages of MI. Notably, at the end stage of MI, no significant pathways were detected by GSEA as the number of DEGs was dramatically reduced, as shown in Fig. 1B.

### Integrated analysis of transcriptome and miRome in the progression of MI

**Figure 2:**
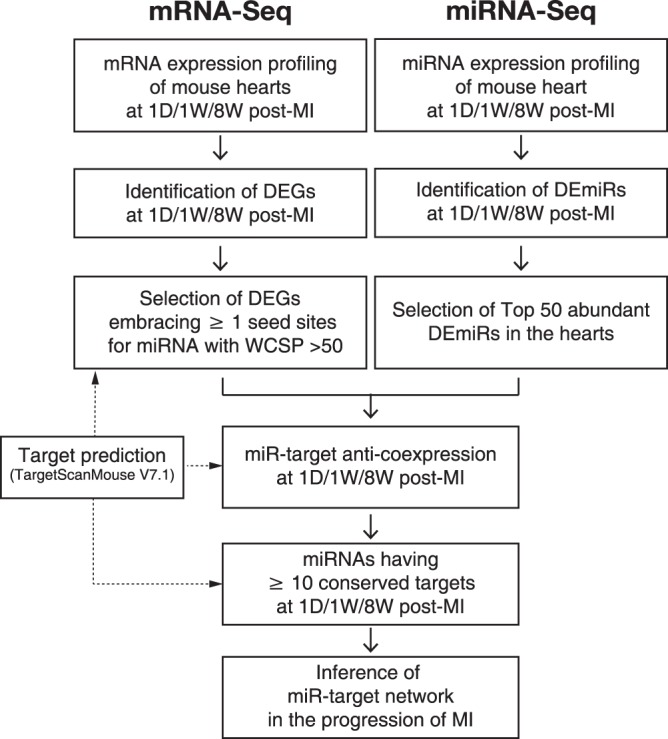
Workflow of inference of miR-target network in MI.

**Figure 3:**
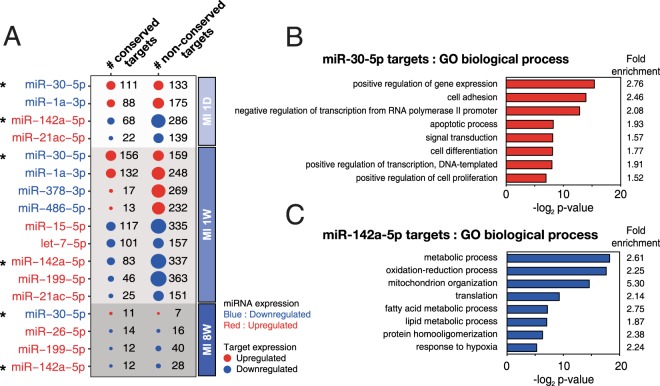
Bubble plot showing differentially expressed miRNAs and their predicted targets and GO enrichment analysis of targets of *miR-30-5p* and *miR-142a-5p*. (**A**) Bubble plot showing differentially expressed miRNAs and their predicted targets showing reciprocal expression at 3 different stages of MI. Red bubble/text indicates upregulation and blue bubble/text denotes downregulation. Note that the expression of the *miR-30-5p* family was significantly downregulated (fold change <2, adjusted *p*-value < 0.05) in all stages of MI, while the expression of *miR-142a-5p* was significantly upregulated (fold change >2, adjusted *p*-value < 0.05) in all stages of MI. (**B**,**C**) Gene Set Enrichment Analysis (GSEA) of *miR-30-5p* family targets (**B**) or *miR-142a-5p* family targets (**C**) that were differentially expressed in 1 day, 1 week, or 8 weeks post-MI. GO analysis was performed using DAVID bioinformatics resources 6.8 (https://david.ncifcrf.gov/).

Since miRNAs are small non-coding RNAs that are not translated into protein, their functions are solely represented through their antagonistic effects on the target genes. In the present study, using both DEG and DEmiR data (Fig. 1), miR-target networks with miRNAs and targets involved in the pathogenesis of MI were identified (Fig. 2). To achieve this, only the top 50 highly expressed DEmiRs, accounting for ~96% of the total miRNA expression, were considered (Supplementary Fig. 6B) to identify the miRNA species that had a significant effect on the pathogenesis of MI. Furthermore, only conserved miRNAs existing across 3 different mammalian species (mouse, rat, and human) were considered due to their evolutional robustness. In addition, to reduce the number of false-positives during target prediction, target genes containing at least one seed site for each miRNA with a weighted context score++ percentile (WCSP) greater than 50 were considered (the WCSP was provided by TargetScan (Ver 7.1) algorithm^7^). Next, we only considered miRNAs that had counterpart DEGs at all stages of MI (Fig. 3). After this stringent screening process, shown in Fig. 2, only 10 miRNAs were left for further study.

**Figure 4:**
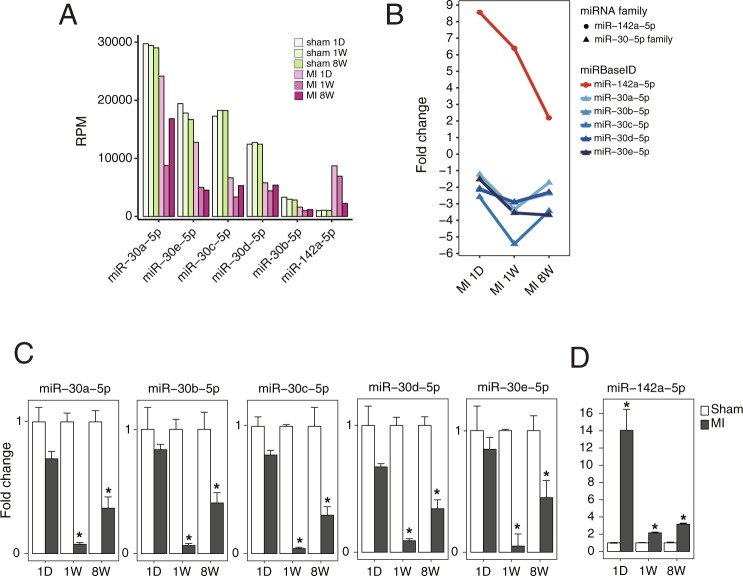
RNA-Seq and qRT-PCR data show the expression levels of *miR-30-5p* family and *miR-142a-5p* in MI. (**A**) Normalized expression levels of *miR-30-5p* and *miR-142a-5p* were measured and (B) the fold expression changes of *miR-30-5p* family and *miR-142a-5p* are shown on day 1, week 1 and week 8 post-MI, derived by miRNA-seq. (**C,D**) The expression levels of the *miR-30-5p* family and *miR-142a-5p* in MI were determined by qRT-PCR. N = 3, Data are expressed as mean ± SEM. *P < 0.05. RPM, Reads Per Million.

Figure 3 demonstrates a bubble plot representing the DEmiRs under MI and their predicted conserved and non-conserved targets. The plot shows miRNA-target networks consisting of 10 miRNA families and 2,298 inversely correlated targets at the different stages of MI. Finally, miRNAs with at least 10 conserved mRNA targets at each stage of MI were selected to minimize false positives. Among the 10 miRNAs, only *miR-30* and *miR-142-5* satisfied the requirements. The RNA-Seq results shown in Fig. 4 demonstrate that *miR-30-5p* was significantly downregulated, while *miR-142-5* was significantly upregulated during all stages of MI. It is interesting to note that the list of 10 miRNAs includes previously reported MI-associated miRNAs, including *miR-1/206*^8^, *miR-21*^9–11^, *miR-15*^12,13^, *miR-199*^14^, and *miR-26*^15^ (Fig. 3A).

### GSEA of the putative targets of *miR-30-5p and miR-142a-5* identifies apoptosis as an important biological process associated with MI

To identify the essential signalling pathways associated with *miR-30-5p* and *miR-142a-5* in the pathogenesis of MI, GSEA was performed on the predicted targets of the miRNAs. Both conserved and non-conserved targets were used for the GSEA analysis, due to the insufficient number of conserved targets for the analysis. The GSEA results showed that apoptosis is an essential GO term associated with MI for *miR-30-5p*. Apoptosis is also an important GO term for *miR-142a-5*, because perturbation of the mitochondrial functions is associated with apoptosis. Apoptosis and necrosis are the two main cell death mechanisms associated with MI, but in our GSEA data, only the apoptosis related GO terms are highly ranked. Hence, we decided to examine apoptosis as a major biological process for both miRNAs.

### qRT-PCR verification of the expression levels of *miR-30-5p* and *miR-142a-5p*

**Figure 5:**
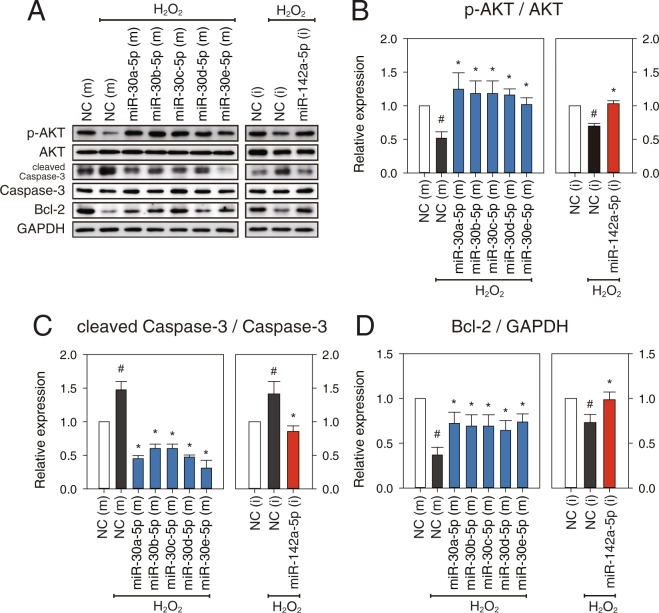
*miR-30-5p* (family) and *miR-142a-5p* regulate apoptotic signalling. At 48 h after transfection with *miR-30-5p* mimics or *miR-142a-5p* inhibitors (50 nM each), NRVMs were treated with 500 μM H_2_O_2_ for 18 h. (**A**) p-AKT, AKT, cleaved Caspase-3, Caspase-3, and Bcl-2 were analyzed by western blotting. GAPDH were used as a loading control. (**B–D**) The expression levels of p-AKT, cleaved Caspase-3, and Bcl-2 were measured. p-AKT and cleaved Caspase-3 were normalized by their total form, AKT and Caspase-3, respectively. Bcl-2 was normalized by GAPDH. Results are presented as mean ± SEM; N = 5; Statistical significance is shown as ^#^*P* < 0.05 relative to NC (m) or *P < 0.05 relative to NC (m) treated with H_2_O_2_. Data were statistically analyzed by one-way ANOVA. Full-length blots/gel are presented in Supplementary Fig. 12.

Figure 4A shows the RNA-Seq-derived expression levels of downregulated *miR-30-5p* and upregulated *miR-142-5p* in MI. The *miR-30-5p* family includes *miR-30a-5p*, *miR-30b-5p*, *miR-30c-5p*, and *miR-30d-5p* (Fig. 5A). The *miR-30-5p* family members with the largest number of conserved target genes were reciprocally regulated at each stage of MI with the highest degree of fold change in the middle stage of MI (1 W) (Fig. 4B). On the other hand, among the upregulated miRNAs shown in Fig. 3, only *miR-142a-5p* showed a sustained increase in expression throughout MI and the most abundant targets at the early stage of MI, where its expression reached a maximal level (Fig. 4A,B). Since RNA-seq was performed on the whole heart extracts, there is a limitation to predict whether the changes in the miRNA and mRNA are also changed in the myocytes. To solve the limitation, the *in vitro* cardiomyocytes model was also used to examine the expression levels of the transcripts. An qRT-PCR analysis validated the expression levels of the *miR-30-5p* family and *miR-142a-5p* both in *in vivo* (Fig. 4C,D) and *in vitro* models of MI (Supplementary Fig. 8). On the basis of the verification results, *miR-30-5p* and *miR-142a-5p* were subjected to further investigation. *miR-30c-5p* was selected as a representative member of the *miR-30-5p* family, since its expression level showed the most dramatic downregulation during the progression of MI and there were no additive effects after the addition of other family members in terms of cardiomyocyte survival (data not shown).

### *miR-30c-5p* (as a representative member of *miR-30-5p* family) and *miR-142a-5p* regulate myocardial apoptosis

To test whether *miR-30-5p* and *miR-142a-5p* regulate myocardial apoptosis at the cellular level, neonatal rat ventricular myocytes (NRVMs) were treated with 500 μM H_2_O_2_ for 18 h after transfection of mimics for *miR-30-5p* or inhibitor for *miR-142a-5p* to overexpress or downregulate the expression of each miRNA. Immunoblot analyses showed that overexpression of *miR-30-5p* family members or inhibition of *miR-142a-5p* significantly upregulated phosphorylation of AKT (p-AKT) and Bcl-2 protein expression (Fig. 5A,B,D). However, the proteolytic cleavage of caspase-3 was significantly reduced by transfection of mimics for the *miR-30-5p* family or inhibitor for *miR-142a-5p* compared to the control group (Fig. 5A,C). The results suggest that normalization of the expression of the *miR-30-5p* family or *miR-142a-5p* during MI inhibits apoptotic signalling but induces cell survival signalling.

**Figure 6:**
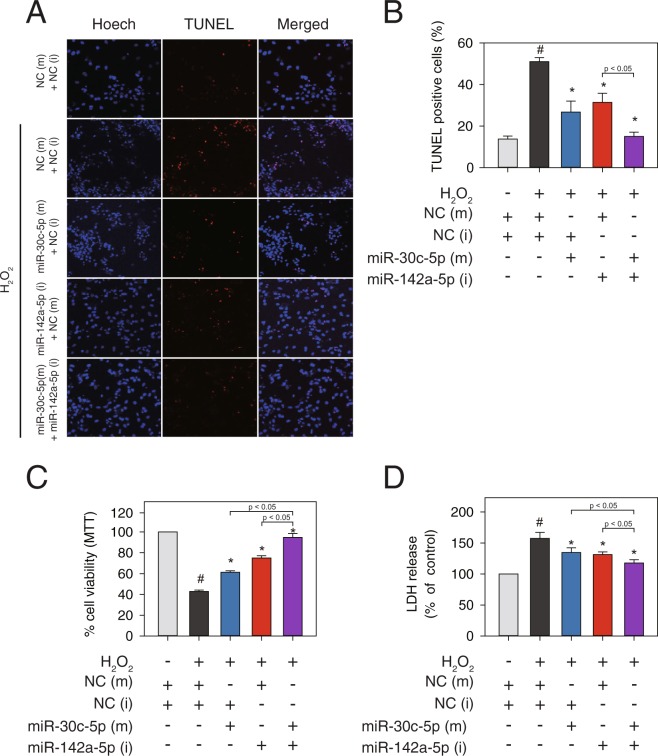
*miR-30c-5p* and *miR-142a-5p* regulate cardiomyocyte apoptosis. At 48 h after transfection with *miR-30-5p* mimics, *miR-142a-5p* inhibitors, or negative controls (25 nM each), respectively or combined, NRVMs were treated with 500 μM H_2_O_2_ for 12 h. (**A**,**B**) Fragmentation was detected by TUNEL assay and cells were counterstained with Hoechst (nuclei staining). (**C**) Cell viability was measured using the MTT (3-(4,5-Dimethyl-2-thiazolyl)-2,5-diphenyl-2H-tetrazolium bromide) assay. (**D**) Cell cytotoxicity was measured using the LDH (lactate dehydrogenase) release assay. Results are presented as mean ± SEM; N = 5–7; Statistical significance is shown as ^#^*P* < 0.05 relative to NC (m) or *P < 0.05 relative to NC (m) treated with H_2_O_2_.

To further examine the anti-apoptotic role of the *miR-30-5p* family and the pro-apoptotic function of *miR-142a-5p*, we conducted TUNEL assays and also measured cell viability with two enzyme release assays. The number of TUNEL-positive cells significantly increased in the H_2_O_2_ treated group, which was then diminished either by overexpression of *miR-30c-5p* mimics or inhibition of *miR-142a-5p* but not by NC (Fig. 6A,B). NRVMs exposed to 500 μM H_2_O_2_ for 12 h showed a markedly decreased viability as assessed by MTT and lactate dehydrogenase (LDH) assays (Fig. 6C,D). In contrast, compared with the NC transfection group, overexpression of *miR-30c-5p* or inhibition of *miR-142a-5p* significantly increased the cell viability of NRVMs treated with H_2_O_2_ (Fig. 6C,D).

*miR-30-5p* (family) and *miR-142a-5p* are reciprocally regulated in the progression of MI and therefore regulate non-overlapping targets during cardiomyocyte apoptosis. We further examined the possible synergistic effects of co-transfection of *miR-30c-5p* mimics and *miR-142a-5p* inhibitor on cardiomyocyte survival. The results showed that the combined treatment of mimics for *miR-30c-5p* and inhibitor for *miR-142a-5p* exerted synergistic effects on cardiomyocyte survival as determined by MTT assay and LDH assay (Fig. 6C,D). Less TUNEL-positive cells were also observed when *miR-30-5p* mimics and *miR-142a-5p* inhibitor were co-transfected compared with that of individual miRNA transfection (Fig. 6A,B). Taken together, our data demonstrates that combinatorial treatment of miRNA mimics for *miR-30c-5p* and inhibitor for *miR-142a-5p* led to a greater protective effect against cardiomyocyte apoptosis, compared with the individual treatments.

### *miR-30c-5p* directly targets 3′-UTR of *Picalm* and *Skil*, and *miR-142a-5p* directly targets 3′-UTR of *Ghr* and *Kitl*

**Figure 7:**
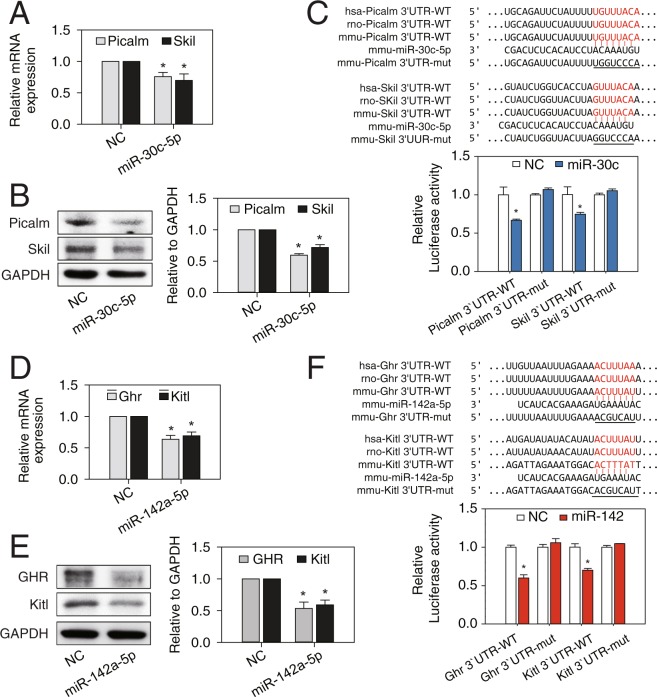
Target validation of *miR-30c-5p* and *miR-142a-5p*. NRVMs were treated with *miR-30c-5p* mimics, *miR-142a-5p* mimics, or negative controls (NC) (50 nM each) for 72 hr. (**A**,**B**) mRNA and protein expression of targets (*Picalm* and *Skil*) of *miR-30c-5p* were significantly decreased. (**C**) Luciferase assay was performed in HEK-293 cells with vectors containing wild type or mutant target 3′UTR, transfected with *miR-30c-5p* or a negative control (NC). (**D**,**E**) mRNA and protein expression of targets (*Ghr* and *Kitl*) of *miR-142a-5p* were significantly decreased. (**F**) Luciferase assay was performed in HEK-293 cells with vectors containing wild type or mutant target 3′UTR, transfected with *miR-142a-5p* or a negative control (NC). Results are presented as mean ± SEM; N = 3; Statistical significance is shown as *P < 0.05 relative to NC. Full-length blots/gel are presented in Supplementary Fig. 13.

Since the mechanism by which miRNAs regulate pathogenesis of MI is entirely dependent on their mRNA targets, we further investigated the direct mRNA targets of these miRNAs. TargetScan analysis initially identified 4,484 and 5,076 genes as putative targets for *miR-30-5p* (family) and *miR-142a-5p*, respectively. After filtering out the genes with a WCSP less than 50 for their binding site at 3′-UTR, 2,908 and 4,266 genes were left for further screening for *miR-30-5p* and *miR-142a-5p*, respectively. Next, due to their anti-co-expressional nature, we considered only 447 upregulated DEGs for *miR-30-5p*, and 531 downregulated DEGs for *miR-142a-5p*. Then, we obtained the intersection between *miR-30-5p* targets and the upregulated DEGs with GO term “positive regulation of apoptotic process” (n = 9) and the intersection between *miR-142a-5p* targets and the downregulated DEGs with GO term “negative regulation of apoptotic process” (n = 4). By doing so, we were finally left with 9 pro-apoptotic genes as candidates for *miR-30c-5p* targets and 4 anti-apoptotic genes as candidates for *miR-142a-5p* targets (Supplementary Fig. 9). The 13 candidate genes were further subjected to verification by qRT-PCR. The qRT-PCR results showed that among 9 genes tested for *miR-30c-5p*, mRNA expression levels of only phosphatidylinositol-binding clathrin assembly protein (*Picalm*) and ski-like proto-oncogene (*Skil)* genes were significantly reduced by overexpression of *miR-30c-5p* (Fig. 7A). Likewise, among 4 genes for *miR-142a-5p*, mRNA expression levels of only growth hormone receptor (*Ghr*) and kit ligand (*Kitl*) genes were significantly reduced by overexpression of *miR-142a-5p* (Fig. 7D). The mRNA and protein expression levels of the identified targets for *miR-30c-5p* and *miR-142a-5p* in MI animal samples also showed similar results (Supplementary Fig. 10). This suggests that *Picalm* and *Skil* are target genes for *miR-30c-5p*, and *Ghr* and *Kitl* are target genes for *miR-142a-5p*.

To determine whether these identified genes are directly targeted by *miR-30-5p* or *miR-142a-5p*, we cloned the wild type and mutated sequence of 3′-UTR of each gene into the 3′-UTR of a luciferase reporter. In the presence of the wild type *Picalm* 3′-UTR or *Skil* 3′-UTR, *miR-30c-5p* repressed the luciferase activity (Fig. 7C). *miR-142a-5p* was also found to decrease the luciferase activity with *Ghr* 3′-UTR or *Kitl* 3′-UTR (Fig. 7F). However, this repression was abolished by a mutation of each miRNA binding site (Fig. 7C,F). Western blotting results also confirmed that protein expression levels of *Picalm* and *Skil* or *Ghr* and *Kitl* were negatively regulated by *miR-30c-5p* and *miR-142a-5p*, respectively (Fig. 7B,E). Taken together, the results suggest that *miR-30c-5p* and *miR-142a-5p* directly bind to 3′-UTR of their targets, resulting in suppressed protein expression.

### siRNA study showed evidence that *miR-30c-5p* directly targets 3′-UTR of *Picalm* and *Skil*, and *miR-142a-5p* directly target 3′-UTR of *Ghr* and *Kitl*

**Figure 8:**
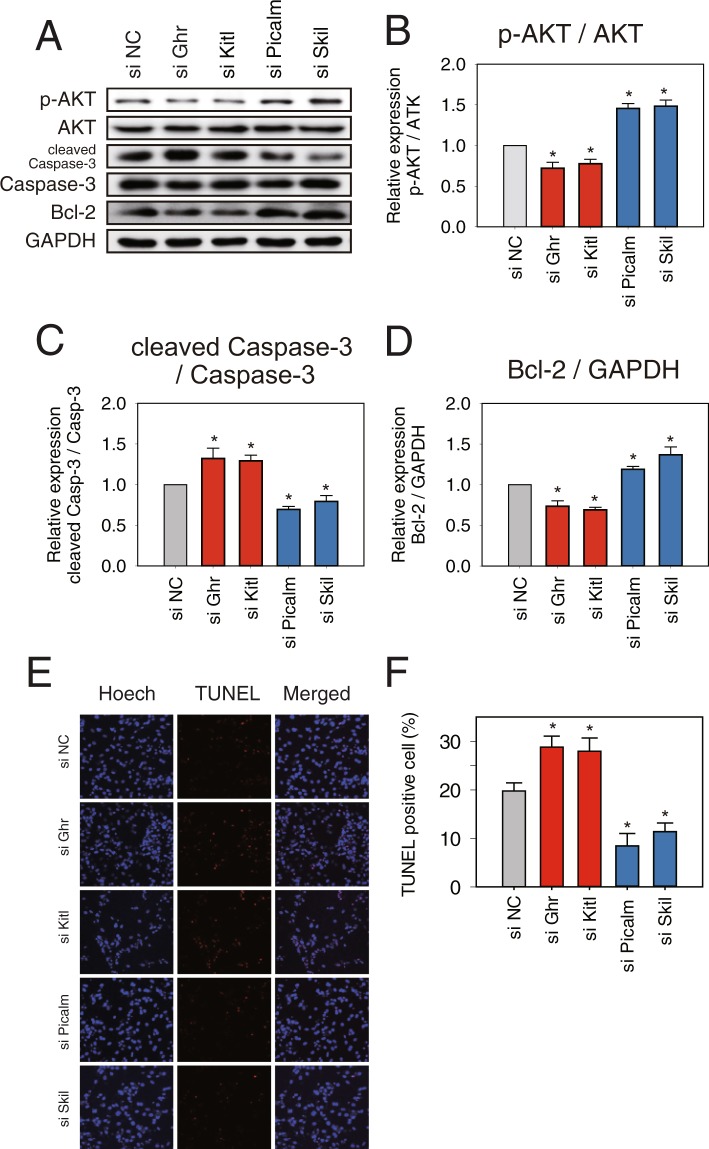
siRNA knockdown of *Picalm*, *Skil*, *Ghr*, and *Kitl* affects cardiomyocyte apoptosis. NRVMs were transfected with *siGhr*, *siKitl*, *siPicalm*, *siSkil*, and siRNA negative controls (siNC) (50 nM each) (**A**) p-AKT, AKT, cleaved caspase-3, caspase-3, and Bcl-2 were analyzed by western blotting. GAPDH was used as a loading control. (**B–D**) The expression levels of p-AKT, cleaved caspase-3, and Bcl-2 were measured. p-AKT and cleaved caspase-3 were normalized by their total form, AKT and caspase-3, respectively. Bcl-2 was normalized by GAPDH. (**E,F**) Fragmentation was detected by TUNEL assays and the cells were counterstained with Hoechst (nuclei staining). Results are presented as mean ± SEM; N = 5; Statistical significance is shown as *P < 0.05 relative to siNC. Data were statistically analyzed by one-way ANOVA. Full-length blots/gel are presented in Supplementary Fig. 14.

To further verify the pro-apoptotic functions of *Picalm* and *Skil* and the anti-apoptotic functions of *Ghr* and *Kitl*, siRNA knockdown experiments were conducted using NRVMs. As described in “Materials and Methods”, siRNA was constructed considering the base sequence of each target mRNA and transfected with 50 nmol/L for 72 hours. The immunoblot results showed that knockdown of *Ghr* (44.9%) or *Kitl* (40.2%) significantly decreased the expression levels of p-AKT 27.8% and 22.3%, respectively and Bcl_2_ 26.7% and 31.1%, respectively, and significantly increased the expression levels of cleaved caspase-3 32.2% and 29.3%, respectively. On the other hand, knockdown of *Picalm* (27.7%) and *Skil* (70.0%) significantly upregulated p-AKT 45.7% and 48.4%, respectively and Bcl-2 18.7% and 36.9%, respectively, but significantly downregulated expression of cleaved caspase-3 30.6% and 20.6%, respectively (Fig. 8A–D, and Supplementary Fig. 11). Furthermore, according to our TUNEL assays, the number of TUNEL-positive cells increased 46.0% and 41.2% by knockdown of *Ghr* and *Kitl*, respectively, but they decreased 42.8% and 57.5% by knockdown of Picalm and Skil, respectively (Fig. 8E,F). The results of the siRNA experiments showed a similar pattern to the ones from the miRNA transfection study, further suggesting that *Picalm* and *Skil* or *Ghr* and *Kitl* are direct targets of *miR-30c-5p* and *miR-142a-5p*, respectively.

## Discussion

**Figure 9:**
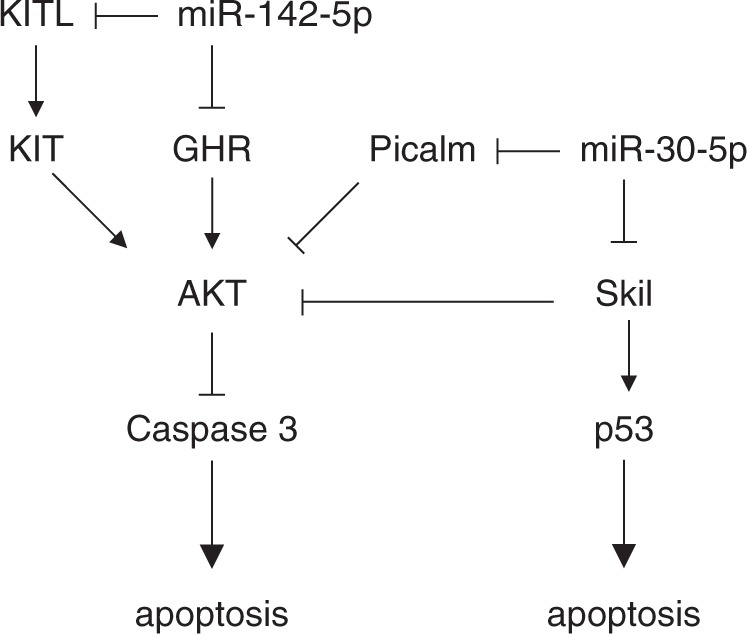
A schematic model to show the effects of *miR-30-5p* and *miR-142a-5p* on apoptosis.

The initiation and progression of MI are associated with complicated and multifaceted pathologies that have been intensely investigated. In the present study, we attempted to identify MI-modulating essential miRNAs and their targets using DEGs and DEmiRs generated from LV samples of sham and MI mice. The novel approach of the present study led to unbiased global DEGs/DEmiRs profiling conducted using sham and MI heart samples (Fig. 1), two-layer omics data integration (Fig. 2) constructing the central miRNA-target anti-co-expressional networks (Fig. 3), identification of anti-apoptotic *miR-30-5p* (family) and pro-apoptotic *miR-142a-5p* involved in MI (Figs 4 and 5), the combined synergistic effects of *miR-30c-5p* mimics and *miR-142a-5p* inhibitor that protects cardiomyocytes from apoptosis (Fig. 6), and the identification of the direct targets of *miR-30-5p* and *miR-142a-5p* (Figs 7 and 8) that might be involved during MI (Fig. 9).

Integration of multilayer-omics data offers an opportunity to understand the flow of genetic information that underlies a certain disease state and can thereby lead to the discovery of their associated disease signatures. This technique has been applied to a wide range of research into aging^16^, microbiology^17^, mitochondrial-related disease^18^, and personalized medicine^19^ in an attempt to identify previously hidden features of disease mechanisms by reducing the gap in our understanding of the relationship between the genotypes and the phenotypes. Recently, it was reported that miRNAs regulate target gene expression mostly through mRNA destabilization with a minor effect on translational efficiency^20^. This suggests that the ultimate impact of miRNAs on their targets can be closely estimated by gene expression profiling using RNA-seq. Therefore, integrated analysis of the transcriptome and miRome generated by simultaneous evaluation of miRNA and target mRNA expression levels in the same biological samples allows for a direct assessment of the reciprocal expression of miRNA and target transcripts, as shown in the present study.

(Supplementary Fig. 6B). The levels of *miR-30-5p* family members have been found to be significantly downregulated in failing rodent and human hearts, leading to interstitial connective tissue growth factor (CTGF)^21^. A previous study also showed evidence that *miR-30* exerts a cardio-protective role against doxorubicin-induced apoptosis^22^. Intriguingly, *miR-30d*, a member of the *miR-30* family, is reported to be synthesized and released into the circulation by cardiomyocytes in response to resynchronization therapy (CRT) in heart failure patients^23^. It has also been reported that higher levels of circulating *miR-30d* are associated with significantly lower mortality in patients with acute heart failure^24^. In this study, we show new evidence that the individual *miR-30-5p* family members are anti-apoptotic in hypoxic conditions (Fig. 6) and that the direct targets of the miRNAs include the two novel apoptotic genes, *Picalm* and *Skil* (Fig. 7A–C). On the contrary, *miR-142a-5p* has been previously reported to be pro-apoptotic in the heart. Its expression was found to be significantly downregulated during cardiac hypertrophy as an adaptive response to changing hemodynamic demand *in vivo*^25^. Forced expression of *miR-142a-5p* had a detrimental effect on heart function while inhibition of *miR-142a-5p* enhanced LV function under sustained p300 signalling. In the model, we show that inhibition of *miR-142a-5p* significantly reduces myocardial apoptosis (Fig. 6), and that the direct targets of *miR-142a-5p* are the two novel anti-apoptotic genes, *KITL* and *GHR* (Fig. 7D–F).

Phosphatidylinositol-binding clathrin assembly protein (PICALM), also known as CALM, is a clathrin assembly protein which recruits clathrin and adaptor protein complex 2 (AP2) to cell membranes at sites of coated-pit formation and clathrin-vesicle assembly. Some recent studies found that *Picalm* is related to apoptosis and that apoptosis was increased in *Picalm*-deficient erythroblasts^26^. SCF-induced KIT trafficking was severely impaired in *CALM*-deficient MEFs leading to the excessive activation of Akt^27^. Knockdown of CALM stimulated autophagosome formation but overexpression decreased autophagy, which protects cells against proapoptotic insults by reducing caspase activation^28^. In the present study, *Picalm* showed proapoptotic effects, as evidenced by the finding that knockdown of *Picalm* increased AKT signalling and decreased cleaved caspase-3 (Fig. 8).

Ski-like proto-oncogene (*Skil)*, also known as *SnoN*, is a member of the Ski family and acts as a negative regulator of transforming growth factor-β (TGF-β). *Skil/SnoN* has both pro- and anti-oncogenic activities in the regulation of cell proliferation, differentiation, and apoptosis, but its function in the heart has not been defined. In Drosophila, *Skil/SnoN* inhibits cell growth when overexpressed^29^. The loss of one allele of *Skil/SnoN* in mice showed a slightly higher rate of tumour formation^30^. *Skil/SnoN* is typically considered to inhibit Smad complexes, and recently published results suggest that *Skil/SnoN* regulates TGF-β signalling with Smad^29,31^. Moreover, *Skil/SnoN* can directly bind and activate p53 to induce apoptosis^32^. In our experiment, a knockdown of *Skil/SnoN* was found to increase survival signalling in neonatal cardiomyocytes (Fig. 9).

Kit ligand (Kitl), also known as stem cell factor (SCF), was reported to bind to the receptor tyrosine kinase c-kit which is expressed in a number of cell types such as cardiac stem cells and adult cardiomyocytes^33^. It activates the Ras/Erk pathway as well as the PI3K-AKT pathway, leading to diverse biological processes such as proliferation, differentiation, and survival^34^. Although c-kit/Kitl signalling is known to be involved in the formation of cardiomyocytes, it has not been thoroughly investigated. C-kit expression is also known to increase as a result of pressure-overload in cardiomyocytes, and cardiomyocyte-specific overexpression of *Kitl* was found to improve cardiac function and survival post-MI^33,35^. In this study, knockdown of *Kitl* showed increased apoptotic signalling (Fig. 8).

It has been suggested that apoptosis is critically linked to the development of heart failure. Growth hormone receptor (GHR) is thought to regulate apoptosis in many cell types including cardiomyocytes through AKT signaling^36,37^. In the present study, we found that GHR was significantly downregulated in the heart in MI (Supplementary Fig. 11) and directly targeted by *miR-142a-5p* (Fig. 7). Knockdown of GHR using siRNA in neonatal cardiomyocytes also showed downregulation of AKT signalling (Fig. 8).

Normalization of *miR-30-5p* and *miR-142a-5p* expression during MI by co-transfection of *miR-30-5p* mimics and *miR-142a-5p* inhibitor resulted in a synergistic protective effect against cardiomyocyte apoptosis in hypoxic conditions, assessed by MTT, LDH, and TUNEL assays (Fig. 6). We attribute this to their completely opposite regulation in MI and the resulting non-overlapping multiple targets in MI-associated signalling pathways.

In conclusion, the present study provided a comprehensive miRNA and mRNA expression profiling in the heart. Integrative analysis of the transcriptome and miRome identified a number of stage-specific miRNA-target networks in MI supported by their reciprocal interactions. Among them, *miR-30-5p* and *miR-142a*, that are oppositely regulated in MI, exerted differential regulation of signalling pathways implicated in the pathogenesis of MI. The combined targeting of *miR-30-5p* and *miR-142a-5p* using proper mimics and inhibitors provided synergistic inhibition of apoptosis under hypoxic conditions. In addition, the use of RNA-Seq data as seen in this study could be beneficial for elucidating miRNA-target networks for other kinds of diseases and thereby for identifying and developing therapeutic tools.

## Materials and Methods

### Ethical statement

All experimental procedures were performed in accordance with the guidelines and regulations approved by the Animal Care and Use Committee of the Gwangju Institute of Science and Technology (IACUC GIST-2017-006) and Chonnam National University (CNU IACUC-H-2016-36).

### Myocardial infarction in mice

MI was induced by permanent ligation of the left anterior descending coronary artery (LAD) as described previously^38^. Briefly, C57BL/6 mice (8 week old males) were subjected to an MI or sham operation under anaesthesia with an intraperitoneal injection of ketamine (50 mg/kg) and xylazine (5 mg/kg). After anesthetization, mice were intubated and mechanically ventilated with room air with the MiniVent Type 845 mouse ventilator (Harvard Apparatus, Holliston, MA, USA). The proximal left anterior descending coronary artery was ligated. Sham-operated animals underwent the same procedure without occlusion of LAD. 1D, 1 W, and 8 W after MI, the mice were sacrificed, and their hearts were removed for further studies.

### Echocardiography to examine the left ventricular function

Left ventricle (LV) functions were assessed by two-dimensional (2D) guided M-mode echocardiography. Echocardiographic studies were performed after anesthetizing the male mice as described above with a 15-MHz linear array transducer system (iE33 system; Philips Medical Systems, Andover, MA, USA). The dimensions of the left ventricular cavity were measured, and the percentage change of the fractional shortening (FS) was calculated as: FS (%) = [(LVEDD − LVESD)/LVEDD] × 100, where LVEDD is LV end-diastolic diameter and LVESD is LV end-systolic diameter. The LV ejection fraction (EF) was calculated as: EF(%) = [(EDV − ESV)/EDV] × 100, where EDV is the LV volume at end-diastole and ESV is the LV volume at end-systole. The volume of the left ventricle was estimated by the area–length method.

### Analysis of RNA-seq data

For RNA-sequencing (RNA-seq), 1 μg of total RNA extracted from pooled mouse LV of MI or sham animals was used to construct cDNA libraries using the TruSeq RNA library kit (Illumina, San Diego, CA, USA) (Supplementary Fig. 1). The libraries were quantified by qPCR according to the qPCR Quantification Protocol (Illumina) and were qualified using an Agilent 2100 Bioanalyzer (Agilent Technologies, Santa Clara, CA, USA). We processed the reads from the sequencer and aligned them to *Mus musculus* (mm10) using Tophat v2.0.13^39^ which incorporates the Bowtie v2.2.3 algorithm^40^. The reference genome sequence of *Mus musculus* (mm10) and annotation data were downloaded from the UCSC table browser (http://genome.uscs.edu). After aligning the reads to the genome, Cufflinks v2.2.1^41^ was used to assemble aligned reads into transcripts and to estimate their abundance. In case of other parameters for Tophat and Cufflinks, default options were used. The relative transcript abundances were measured in FPKM (Fragments Per Kilobase of exon per Million fragments mapped) from Cufflinks. Genes with zeroed FPKM values in all samples were excluded. To facilitate log2 transformation, 1 was added to each FPKM value of filtered genes. Filtered data were log2-transformed and subjected to quantile normalization. Statistical significance of the differential expression data was determined using independent t-tests and fold changes in which the null hypothesis was that no difference exists among groups.

### Analysis of miRNA-seq data

For miRNA-seq, 1 μg of total RNA from the pooled LV of MI and sham was isolated using a miRNeasy Mini kit (Qiagen, Hilden, Germany). The quality and integrity of the RNA samples prepared were verified on a NanoCrop1000 spectrometer (Thermo Scientific, Wilmington, DE, USA) and Bioanalyzer 2100 (Agilent technologies, Santa Clara, CA, USA). Small RNA libraries were prepared using a TruSeq RNA library preparation kit (Illumina, San Diego, USA) in accordance with the manufacturer’s instructions. Briefly, 3′ and 5′ adapters were sequentially ligated to small RNAs, followed by a reverse transcription reaction to create single stranded cDNAs, which were then amplified by PCR and barcoded using a common primer and a primer containing a unique six-base index sequence. The amplified libraries were size-selected/gel-purified and quantified using a Qubit dsDNA HS Assay kit (Life Technologies). Six to eight barcoded libraries were pooled in equimolar (10 nmol/L) amounts and diluted to 8 pmol/L for cluster formation on a single flow cell lane, followed by single-end sequencing on an Illumina HiSeq2000 sequencer. Sequence alignment and detection of known microRNAs were performed using the miRDeep2 algorithm. The preprocessed and clustered reads were aligned to *Mus musculus* matured and precursor miRNAs obtained from miRBase v21. Raw data (the reads for each miRNA) were normalized by the total reads of each individual sample as the standardized to reads per million (RPM, miRNA counts/total counts of each sample × 1 million). We excluded miRNAs with zeroed RPM values across all samples. We added 1 with RPM value of the filtered miRNAs to facilitate log2 transformation. Filtered data were transformed by logarithm and normalized using the quantile normalization method (Supplementary Fig. 1).

### Gene Set Enrichment Analysis (GSEA)

For the identification of enriched transcriptomic signatures, we used the gene set enrichment analysis (GSEA) tool (v3.0) from the Broad Institute at MIT. GSEA was performed by comparison of normalized gene expression data obtained from the 3 different stages of MI. We used hallmark gene sets from MSigDB to interpret the transcriptomic signatures during the progression of MI and performed 1,000 gene set permutations to test for their significance. All basic and advanced fields were set to default. The Enrichment Score (ES) reflects the degree to which a gene set is over-represented at the top or bottom of a ranked list of genes.

### Gene ontology and KEGG pathway analysis

Gene ontology (GO) and Kyoto Encyclopedia of Genes (KEGG) pathway analyses of DEGs and miRNA targets were performed using DAVID 6.7 or Cytoscape with the ClueGO plug-in. The *p*-value was calculated using right-sided hypergeometric tests and the Benjamini-Hochberg correction for multiple testing.

### Primary culture of neonatal rat ventricular myocytes (NRVMs) and transfection

Neonatal rat ventricular myocytes (NRVMs) were isolated from 1-day-old Sprague-Dawley rat pups using a neonatal cardiomyocyte isolation system (Worthington, Columbus, OH, USA), according to the manufacturer’s instructions, as described previously^42^. NRVMs were seeded at a density of 1 million cells per dish onto 1% gelatin-coated 60-mm (Corning, Corning, NY, USA) culture dishes and cultured overnight in DMEM supplemented with 10% FBS, 1% antibiotics (WelGENE, Gyeongsan, Korea), 0.1 mmol/L BrdU at 37 °C in a humidified incubator with 5% CO_2_. The following day, they were placed on a serum-free medium without antibiotics for 24 h prior to miRNA transfection. Cells were then transfected with 50 nmol/L miRNA mimics for miRNA mimics, inhibitors (Dharmacon, Lafayette, CO, USA), or siRNA (Bioneer, Daejeon, Korea) using DharmaFECT-3 reagent (Dharmacon) according to the manufacturer’s instructions. After 48 hours, NRVMs were exposed to of 500 μM H_2_O_2_ for 18 hours. Cells were then harvested for RNA isolation, reverse transcription, qRT-PCR, western blotting, MTT assay, or TUNEL assay.

### Total RNA preparation and reverse transcription

Total RNA and mature miRNAs were isolated from LV or NRVMs using a miRNeasy Mini kit (Qiagen) and reverse transcribed with a miScript Reverse Transcription Kit (Qiagen) in accordance with the manufacturer’s instructions.

### Quantitative real-time PCR (qRT-PCR)

Analysis and quantification of mRNA expression levels were performed with the primers listed in Supplementary Table S3, using SYBR green dye (Kapa Biosystems, Boston, MA, USA) and StepOne Plus Real Time PCR System (Applied Biosystems, Waltham, MA, USA). miRNA-specific qRT-PCR in tissue or isolated cells was performed using a miScript SYBR Green PCR kit (Qiagen) according to the manufacturer’s instructions, with miScript Primer Assay (Qiagen). The expression of mRNAs was normalized to 18S rRNA and the level of mature miRNA was normalized to U6 small RNA using the Hs_RNU6B_2 miScript Primer Assay (Qiagen), respectively. All reactions were performed in triplicate.

### Western blotting

Whole cell lysates were harvested from freshly isolated NRVMs using ice-cold lysis buffer supplemented with a protease inhibitor cocktail (Roche, Basel, Switzerland) and a phosphatase inhibitor cocktail, PhosSTOP (Roche). After determining the protein concentration using a BCA protein assay kit (Pierce), samples were separated by 8–12% SDS-PAGE gel and transferred to polyvinylidene fluoride (PVDF) membranes, followed by blocking with 5% skim milk (BD Life Sciences, Franklin Lakes, NJ, USA) or 5% BSA (Sigma-Aldrich, St. Louis, MO, USA) in TBST (0.1% Tween 20 in Tris-buffered saline; 137 mmol/L NaCl and 20 mmol/L Tris/HCl, pH 7.4) for 1 hour at room temperature (RT), followed by blotting with antibodies, followed by secondary staining with horseradish peroxidase-conjugated immunoglobulin G. Protein expression was detected using an ImageQuant LAS 4000 mini (GE Healthcare Bio-Sciences AB, Uppsala, Sweden). Quantitation was performed with NIH-ImageJ software.

The primary antibodies used were as follows: anti-phospho-AKT (Ser473) (#9271, Cell Signaling, Danvers, MA, USA), anti-AKT (#9272, Cell Signaling), anti-cleaved Caspase-3 (#9661, Cell Signaling), anti-Caspase-3 (#9662, Cell Signaling), anti-Bcl-2 (610539, BD Biosciences, San Jose, CA), anti-Picalm (sc-271224, Santa Cruz, Dallas, TX, USA), anti-Ghr (sc-137185, Santa Cruz), anti-SCF (sc-13126, Santa Cruz), and anti-SnoN (sc-136958 Santa Cruz).

### MTT cell viability assay

Cell viability was measured using the MTT (3-(4,5-Dimethyl-2-thiazolyl)-2,5-diphenyl-2H-tetrazolium bromide) assay. NRVMs were seeded into 96-well plates at a density of 3 × 10^4^ cells/well in DMEM with 10% FBS. After culturing in serum-free DMEM for 24 hours, *miR-30c-5p* mimics, *miR-142a-5p* inhibitors, or a negative control were transfected. Forty-eight hours after transfection, NRVMs were exposed to H_2_O_2_ (500 μM). After 12 hours of incubation, the NRVMs were treated with MTT (5 mg/mL) for 4 hours in the dark at 37 °C. At the end of the incubation period, the untransformed MTT was removed, and dimethylsulfoxide (DMSO) was added to each well to lyse the cells. The plate was then gently rotated to completely dissolve the precipitation. The amount of metabolized MTT was determined by measuring the absorbance at 560 nm using a Victor X3 multi-label plate reader (PerkinElmer, Waltham, MA). The absorbance values were corrected for non-specific conversion of MTT by preparation of blank wells containing all additions apart from the NRVMs. All of the experiments were performed in triplicate. MTT reduction activity was expressed as a percentage of the control.

### LDH cytotoxicity assay

To determine the amount of cardiomyocyte injury induced by H_2_O_2_, the release of lactate dehydrogenase (LDH) was detected. In general, LDH is retained in the cytoplasmic fraction but is released into the surrounding medium when the plasma membrane is ruptured. In the present study, 50 μl of culture medium was taken for the LDH cytotoxicity assay according to the manufacturer’s instruction (Pierce, Thermo Fisher, Waltham, MA, USA) with a microplate reader to measure absorbance at 490 nm.

### TUNEL assay

DNA fragmentation was detected *in situ* using a TUNEL assay kit (*In Situ* Cell Death Detection Kit, TMR red; Roche Applied Science, Penzberg, Germany) as described previously^43^. Briefly, 48 hours after transfection with miRNA mimics or inhibitors for *miR-30a-5p*, *miR-30b-5p*, *miR-30c-5p*, *miR-30d-5p*, *miR-30e-5p*, and *miR-142a-5p*, NRVMs were treated with H_2_O_2_ (500 μM) for 18 h. Cells were then fixed with 4% paraformaldehyde in PBS for 1 h at room temperature, washed with PBS, and permeabilized with 0.2% triton X-100 for 2 min on ice. Nuclear staining was performed with Hoechst (Molecular Probes, Eugene, OR, USA). The percentage of apoptotic nuclei was analyzed using Image J software (NIH Image).

### Luciferase reporter assay

For miRNA target identification, 200–300 base pairs of the 3′-UTR fragments containing the exact target sites for *miR-30-5p* family or *miR-142a-5p* were obtained by PCR amplification. The mutant constructs were generated by standard overlap PCR using mutagenic primers. Next, they were cloned into the NheI and XhoI restriction sites in the pmirGLO dual-luciferase miRNA target expression vector (Promega, Fitchburg, WI, USA). Then, human embryonic kidney (HEK)-293 cells were transfected using Lipofectamin LTX (Invitrogen, Thermo Fisher, Waltham, MA, USA) with 0.5 μg of the pmirGLO chimeric plasmid along with miRNA mimics or inhibitor for the *miR-30-5p* family and *miR-142a-5p* at a final concentration of 50 nmol/L. The luciferase activity was measured using the Dual-Luciferase Reporter Assay System (Promega) on the Victor X3 multi-label plate reader (PerkinElmer, Waltham, MA). Firefly luciferase activity was normalized to Renilla luciferase activity to adjust for variations in transfection efficiency among the experiments.

### Statistical analysis

All data are shown as mean ± standard error of the mean (SEM). For statistical comparison of two groups, an unpaired two-tailed Student’s t-test was used; for multiple comparisons, one-way ANOVA with Bonferroni correction was used. All experiments were performed a minimum of three times. A value of *P* < 0.05 was considered statistically significant.

## Electronic supplementary material

Supplementary materials
