# Supplementary material for: A novel system-level approach using RNA-sequencing data identifies miR-30-5p and miR-142a-5p as key regulators of apoptosis in myocardial infarction
Source: Sci Rep. 2018 Oct 2;8:14638. doi: 10.1038/s41598-018-33020-x (PMC6168573; doi:10.1038/s41598-018-33020-x)
Supplement: Supplementary file 1 — Supplementary materials [file 41598_2018_33020_MOESM1_ESM.docx]

**A novel system-level approach using RNA-sequencing data identifies *miR-30-5p* and *miR-142a-5p* as key regulators of apoptosis in myocardial infarction**

Jin Ock Kim^1,*^, Jei Hyoung Park^1,*^, Taeyong Kim^1,*^, Seong Eui Hong^1^, Ji Young Lee^1^, Kyoung Jin Nho^1^, Chunghee Cho^1^ , Yong Sook Kim^2^, Wan Seok Kang^2^, Youngkeun Ahn^2^ and Do Han Kim^1^

^1^School of Life Sciences and Systems Biology Research Center,

Gwangju Institute of Science and Technology (GIST), Gwangju 61005, Korea

^2^Department of Cardiology, Chonnam National University Hospital, Gwangju, Korea

^*^These authors contributed equally to this work.

**Keywords:** cardiac diseases**,** hypoxia, microRNA, RNA-seq, systems biology, transcriptome, miRome

Correspondence should be addressed to Do Han Kim

(School of Life Sciences, GIST, 123 Cheomdangwagi-ro, Buk-gu, Gwangju, 61005, Korea, E-mail: dhkim@gist.ac.kr, Tel: +82-62-715-2485, Fax: +82-62-715-3411)

Supplementary Information

**Supplementary Figures**

Supplementary Figure. 1


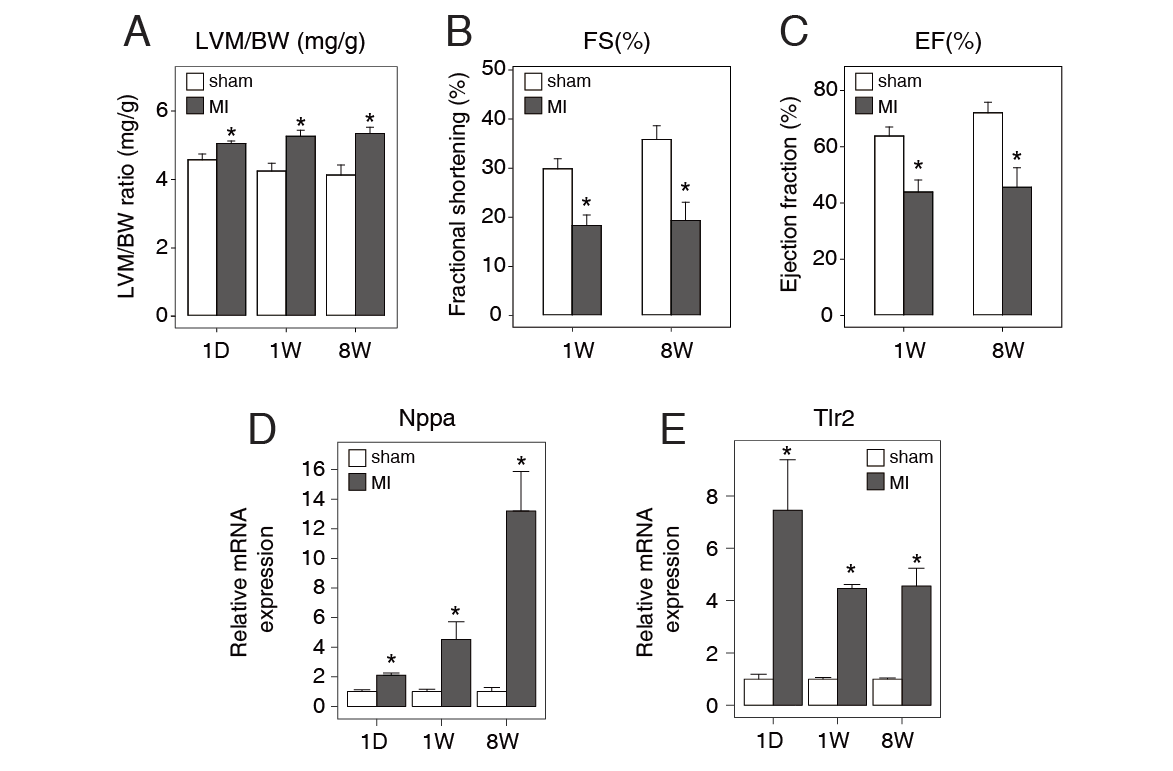


**Supplementary Fig. 1. Generated mouse model of MI.** Cardiac function following MI was assessed 1 day, 1 week, and 8 weeks post-MI. (A) While LV mass (LVM)-to-body weight (BW) ratio (LVM/BW) increased, (B) fractional shortening (FS) and (C) LV ejection fraction (EF) decreased. (D,E) A significant increase in MI marker gene expression, e.g. *Nppa* and *Tlr2*, was also observed. Data are mean ± SEM (n=3). * *P* <0.05 compared to sham.

Supplementary Figure. 2


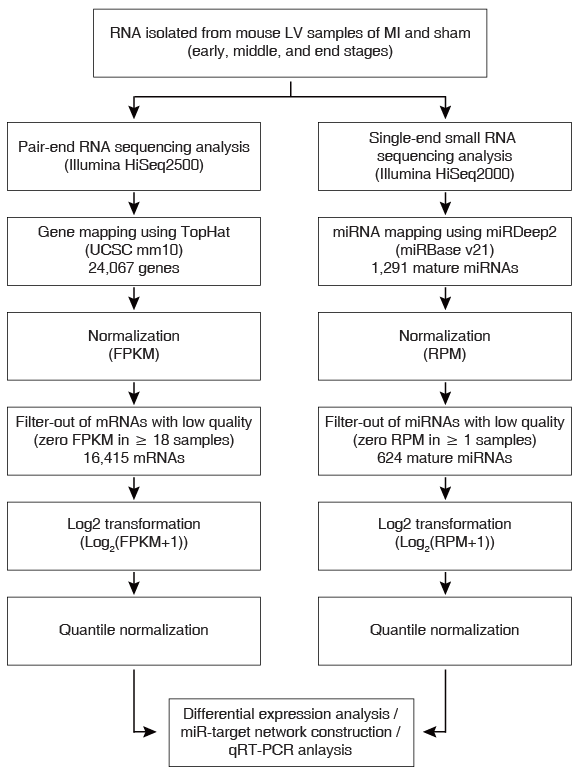


**Supplementary Fig. 2. A workflow of the comprehensive quantification of mRNAs and miRNAs in mouse LV**

Supplementary Figure. 3


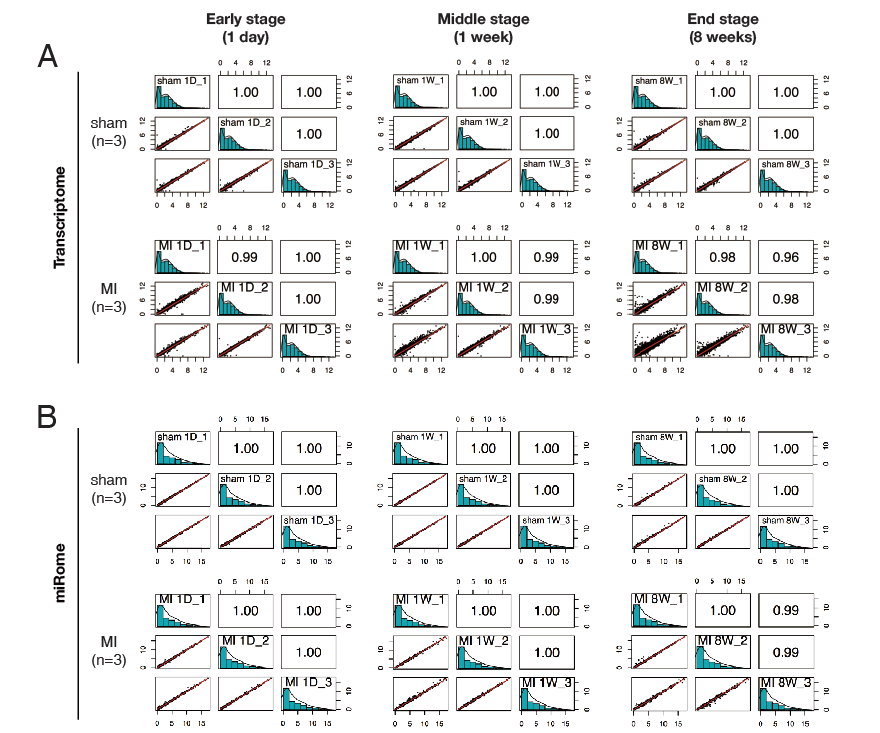


**Supplementary Fig. 3. RNA-seq reveals the reproducibility between the biological replicates.** (A) Analysis of total RNA-seq between replicates and (B) small RNA-seq. Normalized gene expression was expressed as log2(FPKM + 1). In each indicated stage, the lower off diagonal panel shows scatter plots between biological replicates (sample 1 vs. sample 2, sample 1 vs. sample 3, sample 2 vs. sample 3). The diagonal plot shows histograms and the upper off diagonal boxes indicate the correlation constant (R^2^) between biological replicates.

Supplementary Figure. 4


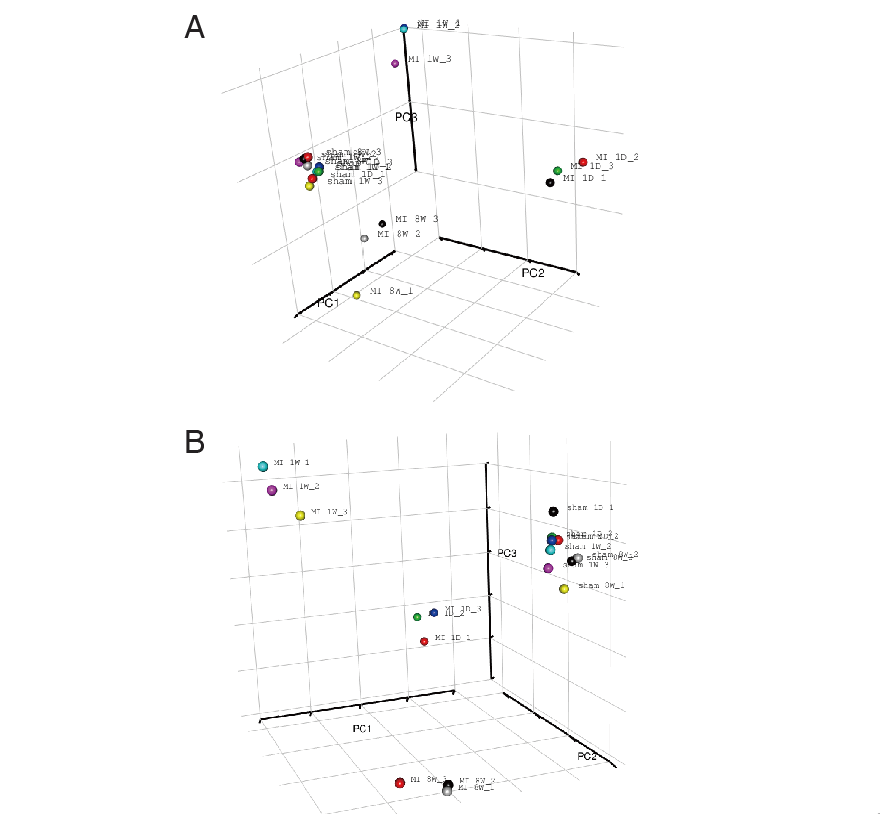


**Supplementary Fig. 4. Three-dimensional principal component analysis.** (A) Analysis based on the data of global mRNA and (B) miRNA expression profiles. mRNA and miRNA expression signatures distribute the LV samples into 3 distinct stages post-MI.

Supplementary Figure. 5


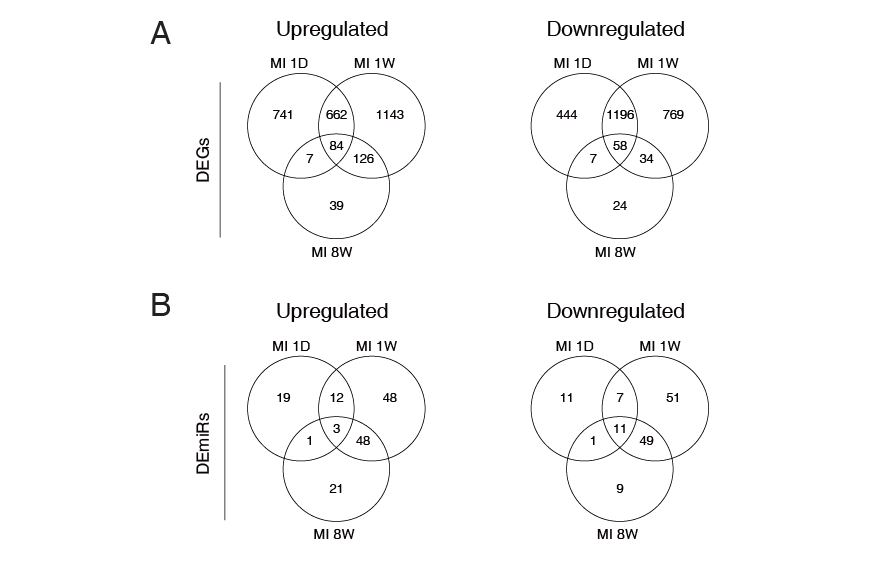


**Supplementary Fig. 5. Venn diagram showing overlaps in DEGs and DEmiRs between the 3 different stages post-MI.**

Supplementary Figure. 6


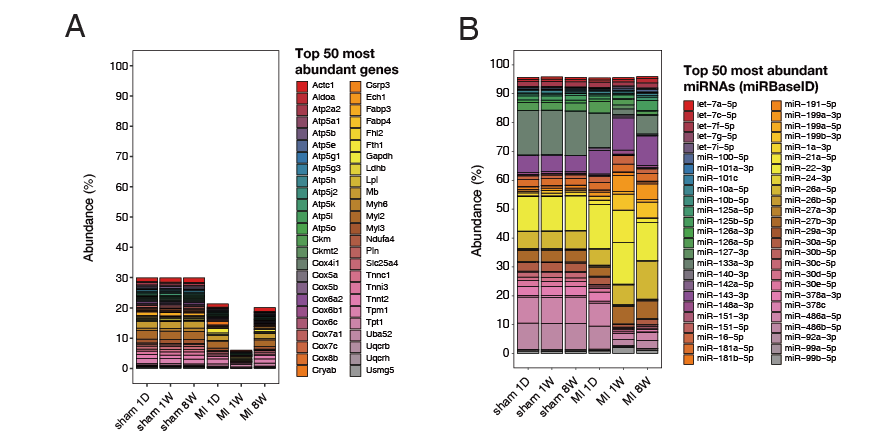


**Supplementary Fig. 6. The percentage of the top 50 highly expressed miRNAs and mRNAs among the total miRNA and mRNA reads in mouse LVs.** (A) The top 50 expressed miRNAs account for ~96% of the total miRNA expression. (B) The top 50 expressed mRNAs account for ~30% of the total mRNA expression.

Supplementary Figure. 7


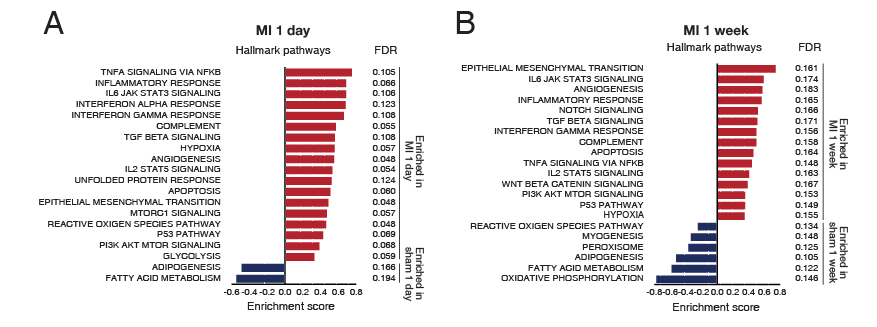


**Supplementary Fig. 7. Global gene expression analysis by GSEA.** (A,B) Gene set enrichment analysis (GSEA) of hallmark gene sets from the Molecular Signatures Database (MSigDB) of the Broad Institute, showing the most significantly enriched gene sets in MI and sham, and their enrichment scores.

Supplementary Figure. 8


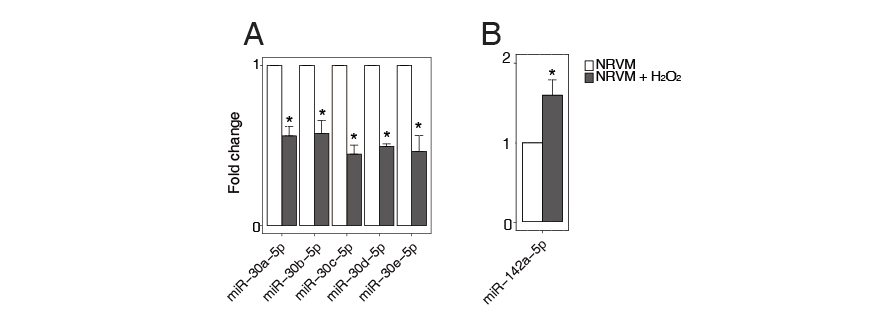


**Supplementary Fig. 8. The miRNA expression in NRVMs.** (A,B) The expression levels of *miR*-*30-5p* (family) and *miR-142a-5p* in cardiomyocytes treated with 500 μM H_2_O_2_ for 18 h. N = 3, Data are expressed as mean ± SEM. **P* <0.05. Data were statistically analyzed by one-way ANOVA

Supplementary Figure. 9


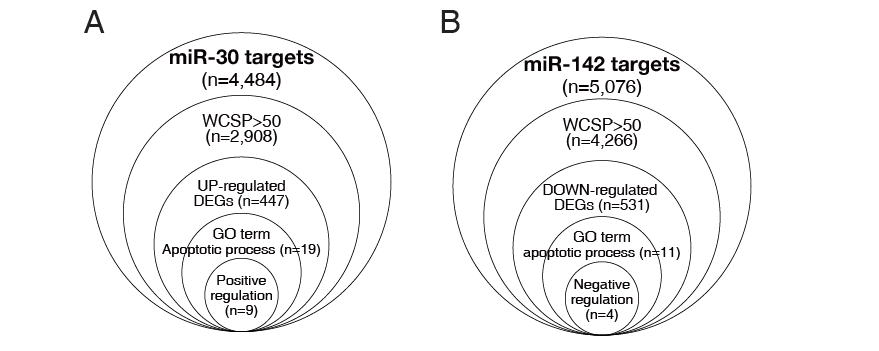


**Supplementary Fig. 9. Identification steps of the *miR-30-5p* and *miR-142a-5p* targets.** To choose targets that regulate apoptosis in MI, weighted context score++ percentile (WCSP), anti-co-expression GO terms were considered.

Supplementary Figure. 10


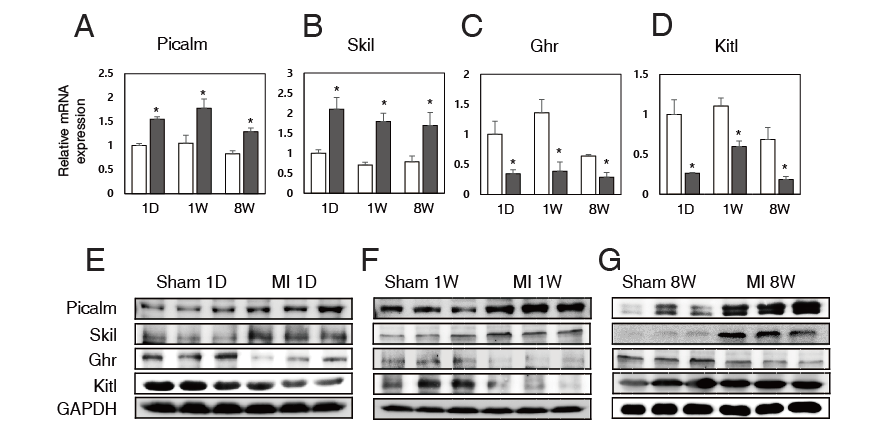


**Supplementary Fig. 10. Expression levels of the *miR-30* and *miR-142* targets in MI.** The expression levels of the *miR-30-5p* and *miR-142a-5p* targets were measured in the MI model by qRT-PCR. (A,B) The mRNA expression of *Picalm* and *Skil*, targets of *miR-30-5p*, is significantly upregulated in day 1, week 1, and week 8 post-MI. (C,D) The mRNA expression of *Ghr* and *Kitl*, targets of *miR-142a-5p*, is significantly downregulated in day 1, week 1, and week 8 post-MI. (E-G) Western blot for *Picalm*, *Skil*, *GHR*, and *Kitl* in the hearts of sham and MI mice. Note that the western data (E-G) are in agreement with the PCR data (A-D), except for the no significant change of Kitl shown in 8W MI. It could be caused by an unidentified modification of protein expression. Data are mean ± SEM (N=3). * *P* <0.05 compared to sham.

Supplementary Figure. 11


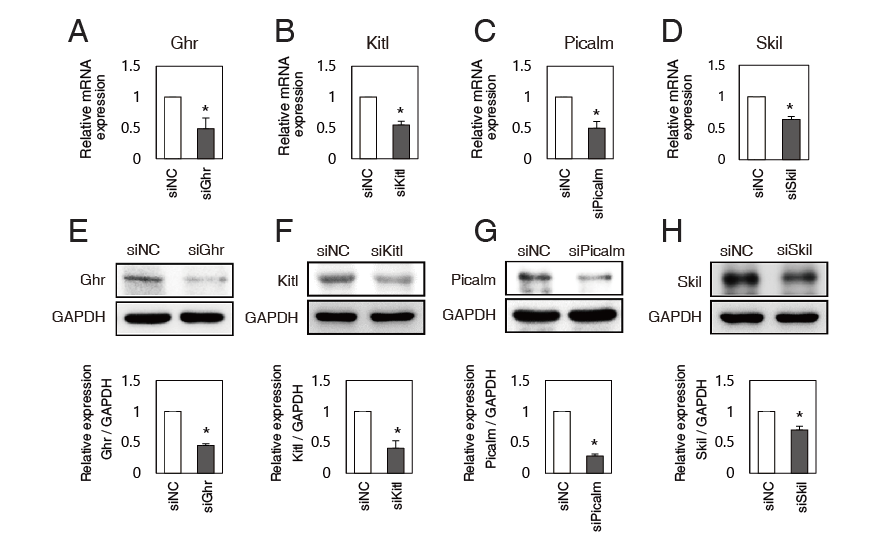


**Supplementary Fig. 11. si-mediated knockdown of the target proteins and mRNAs for *miR-30* and *miR-142*.** NRVMs were transfected with *siGhr*, *siKitl*, *siPicalm*, *siSkil*, or siRNA negative control (siNC) (50 nM each). (A-D) each mRNA expression was measured by qRT-PCR. (E-H) Protein expression was measured by western blotting. Data are expressed as mean ± SEM; Statistical significance is shown as **P* <0.05. Data were statistically analyzed by one-way ANOVA.

Supplementary Figure. 12


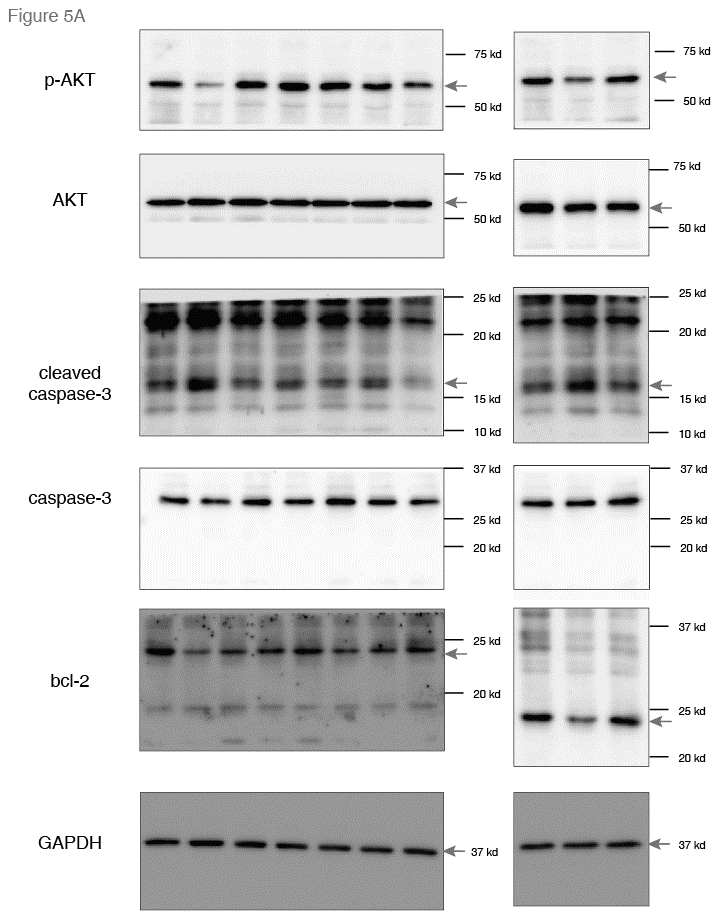


Supplementary Figure. 13


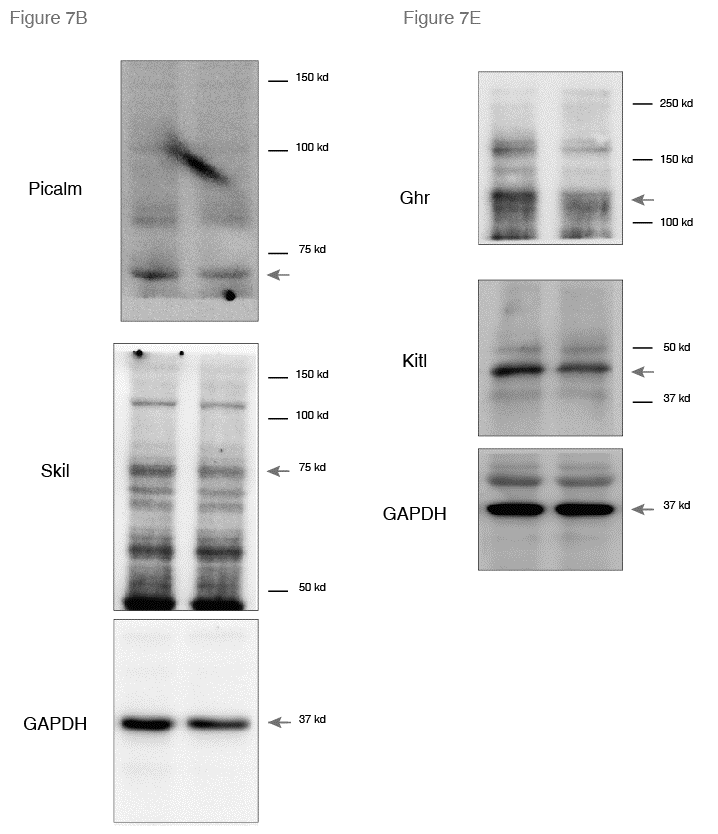


Supplementary Figure. 14


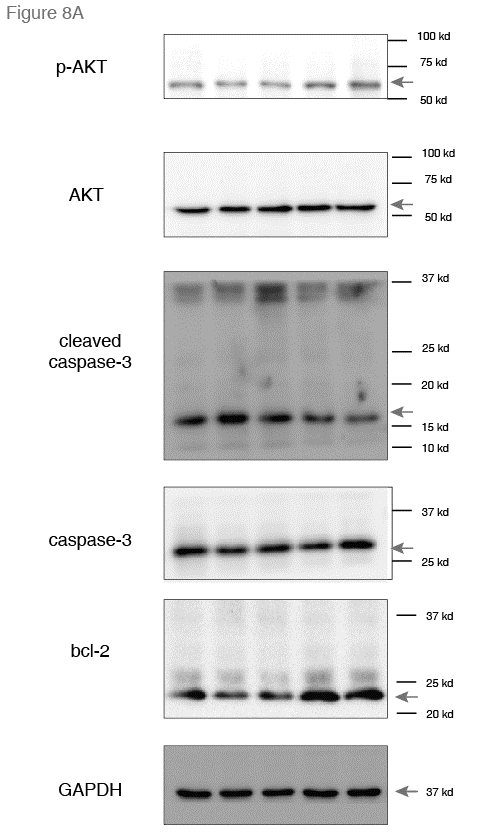


**Supplementary Tables**

**Supplementary Table 1. Summary of total RNA-seq read count and mapping results**

|  | **Sample** | **Read**  **Type** | **Total processed reads** | **Mapped Reads (UCSC mm10)** | **Overall  Read Mapping Ratio** |
| --- | --- | --- | --- | --- | --- |
| 1 | sham 1D-1 | 1 | 35425113 | 34530569 (97.5%) | 96.90% |
| 1 | sham 1D-1 | 2 | 35425113 | 34106608(96.3%) |  |
| 2 | sham 1D-2 | 1 | 37666175 | 36677531 (97.4%) | 96.90% |
| 2 | sham 1D-2 | 2 | 37666175 | 36308132 (96.4%) |  |
| 3 | sham 1D-3 | 1 | 37498372 | 36497107 (97.3%) | 96.80% |
| 3 | sham 1D-3 | 2 | 37498372 | 36079176 (96.2%) |  |
| 4 | MI 1D-1 | 1 | 36638058 | 35659710 (97.3%) | 96.80% |
| 4 | MI 1D-1 | 2 | 36638058 | 35287161 (96.3%) |  |
| 5 | MI 1D-2 | 1 | 34399810 | 33507067 (97.4%) | 96.80% |
| 5 | MI 1D-2 | 2 | 34399810 | 33112118 (96.3%) |  |
| 6 | MI 1D-3 | 1 | 40759432 | 39702813 (97.4%) | 96.80% |
| 6 | MI 1D-3 | 2 | 40759432 | 39232880 (96.3%) |  |
| 7 | sham 1W-1 | 1 | 37126141 | 36143899 (97.4%) | 96.90% |
| 7 | sham 1W-1 | 2 | 37126141 | 35796826 (96.4%) |  |
| 8 | sham 1W-2 | 1 | 36813673 | 35836905 (97.3%) | 96.90% |
| 8 | sham 1W-2 | 2 | 36813673 | 35493216 (96.4%) |  |
| 9 | sham 1W-3 | 1 | 37655644 | 36707109 (97.5%) | 96.90% |
| 9 | sham 1W-3 | 2 | 37655644 | 36304271 (96.4%) |  |
| 10 | MI 1W-1 | 1 | 31638788 | 30744167 (97.2%) | 96.30% |
| 10 | MI 1W-1 | 2 | 31638788 | 30182673 (95.4%) |  |
| 11 | MI 1W-2 | 1 | 29509480 | 28718573 (97.3%) | 96.60% |
| 11 | MI 1W-2 | 2 | 29509480 | 28307149 (95.9%) |  |
| 12 | MI 1W-3 | 1 | 35471393 | 34522041 (97.3%) | 96.70% |
| 12 | MI 1W-3 | 2 | 35471393 | 34062917 (96.0%) |  |
| 13 | sham 8W-1 | 1 | 38125733 | 37177671 (97.5%) | 97.10% |
| 13 | sham 8W-1 | 2 | 38125733 | 36833956 (96.6%) |  |
| 14 | sham 8W-2 | 1 | 32433701 | 31617920 (97.5%) | 97.00% |
| 14 | sham 8W-2 | 2 | 32433701 | 31316863 (96.6%) |  |
| 15 | sham 8W-3 | 1 | 34474283 | 33612611 (97.5%) | 97.00% |
| 15 | sham 8W-3 | 2 | 34474283 | 33272386 (96.5%) |  |
| 16 | MI 8W-1 | 1 | 31186056 | 30396508 (97.5%) | 97.00% |
| 16 | MI 8W-1 | 2 | 31186056 | 30107948 (96.5%) |  |
| 17 | MI 8W-2 | 1 | 32298589 | 31489721 (97.5%) | 97.00% |
| 17 | MI 8W-2 | 2 | 32298589 | 31170039 (96.5%) |  |
| 18 | MI 8W-3 | 1 | 31916752 | 31079446 (97.4%) | 96.70% |
| 18 | MI 8W-3 | 2 | 31916752 | 30646597 (96.0%) |  |

**Supplementary Table 2. Summary of small RNA-seq read count and mapping results**

|  | **sample** | **Total processed reads** | **Mapped Reads  (miRBase v21)** | **Overall  Read Mapping Ratio** |
| --- | --- | --- | --- | --- |
| 1 | sham 1D-1 | 29520078 | 24128227 | 81.70% |
| 2 | sham 1D-2 | 30479933 | 24747344 | 81.20% |
| 3 | sham 1D-3 | 30852937 | 26154975 | 84.80% |
| 4 | MI 1D-1 | 17719902 | 8506491 | 48.00% |
| 5 | MI 1D-2 | 21292109 | 10483316 | 49.20% |
| 6 | MI 1D-3 | 24161638 | 11262723 | 46.60% |
| 7 | sham 1W-1 | 28983173 | 23806871 | 82.10% |
| 8 | sham 1W-2 | 29516749 | 24042862 | 81.50% |
| 9 | sham 1W-3 | 29764009 | 24755331 | 83.20% |
| 10 | MI 1W-1 | 30013639 | 23862205 | 79.50% |
| 11 | MI 1W-2 | 26738888 | 20943587 | 78.30% |
| 12 | MI 1W-3 | 28694581 | 22973370 | 80.10% |
| 13 | sham 8W-1 | 26213422 | 22074988 | 84.20% |
| 14 | sham 8W-2 | 28861972 | 24389737 | 84.50% |
| 15 | sham 8W-3 | 29791863 | 25083122 | 84.20% |
| 16 | MI 8W-1 | 24781990 | 19385296 | 78.20% |
| 17 | MI 8W-2 | 32109379 | 27087783 | 84.40% |
| 18 | MI 8W-3 | 28959171 | 23893545 | 82.50% |

**Supplementary Table 3. Table S3. Primers for qRT-PCR analysis**

| **Gene** | **Forward primer (5'-3)** | **Revers primer (5'-3')** |
| --- | --- | --- |
| 18S | TTCTGGCCAACGGTCTAGACAAC | CCAGTGGTCTTGGTGTGCTGA |
| Mmp12 | CTCTAGCCAGCACATGACTCCAA | CTGATGTGAAATGAGCCACACAAC |
| Hp | GCTATCGCTGCCGACAGTTCTAC | TCTCCAGCGACTGTGTTCACC |
| Csf2rb2 | TCAGATGAGGGTCTGCTGTGGTA | AAGGCCATGTGCAAACGTGA |
| Tlr13 | CTGATGAGGCATGGGTATACAAAGA | AAATCCCGTTGGTGAAGACAGAG |
| Cd74 | AGCCAGATGCGGATGGCTA | TCCTGGGTCATGTTGCCGTA |
| Bex1 | CACCTGGTGGTGAGCATCTCTA | GCCTTGATCTTTGGACTCCATTA |
| Adprhl1 | ATAACTACCTCCTCGCGTGG | ATCTCTCTTCCCCACTGCAC |
| Klhl34 | AAGTTGTTCTTGACCTTGCCTGA | TGACCATTCCTGACTGACATTTG |
| Hspb6 | AGCCTAGTCCACCAGTCCTA | TGAGTTTGGGGTTCCTGGTT |
| Myot | AGGTTGTTAGGACCGCAGAATG | CTTGAATGAAGCGTGGTGGGTA |
| Tlr2 | TGTCTCCACAAGCGGGACTTC | TTGCACCACTCGCTCCGTA |
| Picalm | CAACGGGCATGATAGGATATGGA | CAGGCTGGCTGTATATTAAGGTTGG |
| Skil | AGTTTCTAACCTGGGAAGGCA | TGTGTCGTGTAATGCAACCA |
| Kitl | TCTGTCTTGGAGCTGCATGA | TGGGGCTTCATATTGCAGGA |
| Ghr | TCACATGGAGGCCACGACA | TCTCGGACATTCGGGCAGTAG |
| Bnp | CAGCTGCCTGGCCCATCACT | ACCTCCCAGCGGCGACAGAT |
| Anf | ACCTGCTAGACCACCTAGAGG | GCTGTTATCTTCCGTACCGG |
